# Associations between salivary cytokines and oral health, age, and sex in healthy children

**DOI:** 10.1038/s41598-022-20475-2

**Published:** 2022-09-26

**Authors:** Charlotte Rinderknecht, Cornelia Filippi, Nicole Ritz, Nora Fritschi, Urs Simmen, Andreas Filippi, Tamara Diesch-Furlanetto

**Affiliations:** 1 Department of Oral Surgery, University Center for Dental Medicine, University of Basel, Mattenstrasse 40, 4058 Basel, Switzerland; 2 Department of General Pediatric and Adolescent Dentistry, University Center for Dental Medicine, University of Basel, Basel, Switzerland; 3 Mycobacterial and Migrant Health Research Group, University Children’s Hospital Basel and Department of Clinical Research, University of Basel, Basel, Switzerland; 4 Department of Paediatrics and Paediatric Infectious Diseases, Children’s Hospital, Lucerne Cantonal Hospital, Lucerne, Switzerland; 5 Department of Pediatrics, The Royal Children’s Hospital Melbourne, The University of Melbourne, Melbourne, Australia; 6 University Children’s Hospital Basel, University of Basel, Basel, Switzerland; 7 Simmen Statistical Consulting, Basel, Switzerland; 8 Department of Oncology/Haematology, University Children’s Hospital Basel UKBB, Basel, Switzerland

**Keywords:** Cytokines, Dentistry, Paediatrics

## Abstract

Human saliva is a complex fluid containing proteins such as salivary cytokines, which can be used for diagnostic purposes, particularly among the pediatric population. This study aimed to assess the concentrations of salivary cytokines in healthy children and adolescents and determine their associations with age, sex, and oral and dental findings. Healthy children and adolescents aged 4–18 years were enrolled in this cross-sectional study. The concentrations of the following salivary cytokines were measured by Luminex technology: IFN-γ, IL-1α, IL-1β, IL-4, IL-5, IL-6, IL-8, IL-10, IL-13, IP-10, TNF-α, and VEGF-A. Additionally, oral and dental parameters were recorded using a standardized protocol. A total of 128 participants (mean age, 10.7 years; males, 50.8%) were enrolled. The levels of 1β, IL-6, IL-8, and IL-10 were significantly higher in those with gingivitis. Increased salivary flow rates were negatively correlated with IL-1α, IL-1β, IL-6, IL-8, IL-10, TNF-α, and VEGF-A concentrations. The findings of this study showed that the concentrations of most of the salivary cytokines were positively correlated with age and the presence of oral pathologies (such as gingivitis and caries) and negatively correlated with salivary flow rate.

## Introduction

Cytokine concentrations are measurable in several body fluids, tissues, and cells for diagnostic or prognostic purposes^1^. Human saliva is a complex fluid that has gained recent interest as a diagnostic sample to detect cytokines. In pediatrics, the use of blood cytokines for diagnostic purposes has most commonly been studied in systemic diseases such as neonatal sepsis, tuberculosis, pneumonia, and neuroinflammatory as well as rheumatological diseases^2,3^. Salivary cytokines were further assessed as diagnostic biomarkers for oral conditions such as caries, gingivitis, and periodontitis^4–6^.

Contrary to blood, saliva sampling is a noninvasive procedure; moreover, it does not require trained staff and is cost effective. Thus, saliva collection is particularly useful in instances where the collection of blood is challenging, such as in young children and elderly or patients with anxiety^6,7^. Only a few studies have analyzed the salivary cytokines in children, and most of the studies were conducted in children with acute lymphatic leukemia to determine the presence of a possible biomarker for graft versus host disease or mucositis^8–13^. Additionally, a few studies investigated salivary cytokines as markers for caries and gingivitis^14–19^. Data on the concentrations of salivary cytokines in healthy pediatric populations are negligible. In order to find diagnostic biomarkers in the future that can predict oral complications and support diagnosis of systemic diseases as well as treatment efficacy, we depend on reference values in healthy children. The concentrations of cytokines in blood increase with age until early adolescence^20,21^; therefore, we hypothesized that the concentrations of salivary cytokines might be associated with age and oral health.

This study aimed to assess the cytokine concentrations in healthy children and adolescents and assess their association with age, sex, salivary flow rate and oral health. The findings might contribute to our understanding and interpretation of salivary cytokine-associated assays in the pediatric population.

## Materials and methods

### Study population

This prospective observational study comprised healthy children aged 4–18 years who visited the Department of Children and Adolescent Dentistry, University Center for Dental Medicine Basel, University of Basel, Switzerland, for routine dental prophylaxis consultations over a period time of 2 years. The exclusion criteria for this study were as follows: history of acute symptoms of respiratory infections in the preceding 2 weeks (including rhinitis, bronchitis, pharyngitis, cough, and tonsillitis); known allergy to paraffin; hemato-oncological or autoimmune disease; use of systemic antibiotics in the preceding 2 weeks; use of antimicrobial rinsing solutions 12 h before the study; and any vaccination within the preceding 48 h. Written informed consent, parental where required, was obtained for each patient.

Demographic and clinical information were collected from each child in a standardized form using an electronic database (Epidata manager, v.2.0.13.65 and Epidata entry client, v.2.0.10.26 by EpiData Association, Denmark). The following variables were recorded: age; sex; the presence or absence of gingivitis, washable plaque, caries, xerostomia, candidiasis, mucosa blandness, and aphthae and its localization; number of carious, missing, and filled teeth; date of saliva collection; total volume of saliva collected; and the salivary flow rate. All oral findings were assessed and recorded by 7 experienced dentists, who had been previously trained for this study and had obtained detailed information concerning study protocol and procedure. The study was approved by the ethics committee of North–West Switzerland (Number: 2016-00583) and all methods were performed in accordance with the relevant guidelines and regulations.

### Saliva collection

The saliva was collected 30–90 min after eating or brushing the teeth in the morning hours between 8 am and 12 noon before the oral cavity was examined or any dental treatment was provided. The sample was obtained by asking the patients to chew on paraffin gum for 2–5 min while tilting their head forward and repeatedly expectorate the saliva into a cup. The saliva volume was measured (1 g equivalent to 1 ml) and a saliva flow rate (ml/min) was calculated. The samples were stored at 4 °C within 20 min. Subsequently, they were centrifuged at 1650 gravity (G) for 15 min before cryopreservation at − 75 °C; only those with a volume of at least 30 μl were retained for further experiments.

### Cytokine measurement

Salivary cytokines were selected based on previous work by Diesch et al.^6^. In addition, we analyzed other potentially relevant cytokines in order to get a spectrum of cytokines as comprehensive as possible. Batch analyses of the salivary samples were performed using a multiplex cytokine analyzer (Magpix, Luminex Corp. Austin, US) with magnetic bead-based multianalyte panels (Milliplex, Merck Millipore, Schwalbach, Germany). The following cytokines were measured: IFN-γ, interleukin (IL)-1α, IL-1β, IL-4, IL-5, IL-6, IL-8, IL-10, IL-13, IFN-γ-inducible protein (IP)-10, tumor necrosis factor (TNF)-α, and vascular endothelial growth factor (VEGF-A). The samples were thawed, centrifuged, and stained according to the manufacturers’ instructions and the protocol for cell culture supernatants. The concentrations of the cytokines were calculated using a log regression standard curve. If the concentrations were below the quantification limit, they were set to half the detection limit (DL), as in the cases of IFN-γ (DL = 1.3), IL-1β (1.6), IL-13 (6.4), TNF-α (6.4), and IP-10 (2.6).

### Statistical analyses

The patients were stratified into two age groups: 4–11 (children) and 12–18 (adolescents) years. The descriptive statistics included the frequency (proportion), median (interquartile range [IQR]) or mean ± the standard deviation. The corresponding p-values were derived from significance tests such as the Chi-squared test, Fisher’s exact test, Wilcoxon rank-sum test, or t-test. To predict the association between age and the cytokine level, linear regression models adjusted for sex, gingivitis, caries, and salivary flow rate were used. The models had a nested design to receive separate estimates for the two age groups. The cytokine concentrations had to be log-transformed for the regression analysis as verified by quantile comparison plots and “standardized residuals versus fitted values plots.” Additionally, significance tests were performed for potential nonlinear age dependency and other significant interactions. The back-transformed estimates for the predicted cytokines are presented as the ratio per year of age or the geometric mean ratios for sex and health status. Additionally, the corresponding 95% confidence intervals and p-values are indicated. No adjustments to the significance levels for multiple comparisons were made because of the exploratory nature of the study. All analyses were performed using the statistical program R version 3.5.1 (Austria).

## Results

### Demographic and clinical profile

Of the 143 children enrolled in the study, 128 were included in the final analysis according to the described exclusion criteria. The mean age was 10.7 ± 4.2 years; 50.8% of the participants were males, 60.2% were children; and 39.8% were adolescents (Table 1). The median amount of saliva collected was 4.2 ml (IQR: 2.6, 6.5), and the mean salivary flow rate was 1.0 ± 0.7 ml/min. Adolescents had higher median saliva quantity and higher mean salivary flow rate than children. Tables 1 and 2 summarize the additional patient characteristics and baseline cytokine concentrations, respectively.

**Table 1:** Baseline characteristics of the study participants.

	Child n = 77	Adolescent n = 51	Total n = 128	p-value
Males/females^a^	41/36 (53.2/46.8)	24/27 (47.1/52.9)	65/63 (50.8/49.2)	0.61
Age^b^	7.8 (2.4)	15.1 (1.7)	10.7 (4.2)	< 0.001
**Stomatological and dental findings**				
Caries^a^	38 (49.4)	11 (21.6)	49 (38.3)	0.003
Gingivitis^a^	14 (18.2)	21 (41.2)	35 (27.3)	0.008
Washable plaque^a^	51 (66.2)	21 (41.2)	72 (56.2)	0.009
Xerostomia^a^	0 (0)	0 (0)	0 (0)	n.e
Candidiasis^a^	0 (0)	1 (2)	1 (0.8)	0.40
Decayed teeth^c^	0.5 (0, 3)	0 (0, 0)	0 (0, 1.5)	0.001
Missing teeth^c^	0 (0, 1)	0 (0, 0)	0 (0, 0.5)	0.080
Filled teeth^c^	2 (0, 5)	1 (0, 3)	2 (0, 4)	0.27
Any mucosal changes^a^	4 (5.2)	2 (3.9)	6 (4.7)	1
Aphthae^a^	2 (2.6)	0 (0)	2 (1.6)	0.52
Saliva total quantity (ml)^c^	3.2 (2.1, 4.7)	6.0 (4.0, 10.7)	4.2 (2.6, 6.5)	< 0.001
Salivary flow rate (ml/min)^b^	0.8 (0.5)	1.5 (0.8)	1.0 (0.7)	< 0.001

*n.e.* not estimatable.

^a^The values are given as the frequency (and proportion).

^b^The values are given as the mean (and the standard deviation).

^c^The values are given as the median (and the interquartile range).

**Table 2:** Salivary cytokine concentrations in the study population.

	Child n = 77	Adolescent n = 51	Total n = 128	p-value
IFN-γ	1.1 (0.6, 2.7)	1.1 (0.6, 2.5)	1.1 (0.6, 2.7)	0.86
IL-1α	733 (402, 1433)	806 (404, 1534)	756 (398, 1471)	0.60
IL-1β	16.1 (8.1, 34.2)	17.1 (6.9, 41.2)	16.1 (7.7, 36.6)	0.75
IL-4	1.4 (0.9, 2)	1.6 (0.9, 2.1)	1.4 (0.9, 2)	0.40
IL-5	0.9 (0.6, 1.2)	1.1 (0.7, 1.5)	0.9 (0.6, 1.3)	0.032
IL-6	3.3 (1.4, 8.5)	2.6 (1.3, 5.1)	2.9 (1.4, 8.1)	0.39
IL-8	297 (152, 590)	222 (137, 714)	282 (147, 609)	0.66
IL-10	7.6 (4.1, 11.8)	7.6 (4.7, 11.5)	7.6 (4.3, 11.8)	0.92
IL-13	9.0 (3.2, 15.4)	8.6 (3.3, 21.3)	9.0 (3.2, 18.9)	0.40
IP-10	39.6 (16.7, 103)	63.7 (22.7, 112)	52.6 (17.7, 105)	0.35
TNF-α	7.1 (4.5, 12.4)	7.8 (4.4, 13.1)	7.7 (4.4, 13.1)	0.72
VEGF-A	164 (110, 249)	154 (70, 263)	163 (88, 255)	0.39

The values are given as the median (and the interquartile range).

### Salivary cytokines

The median concentrations of the 12 cytokines had a range from 0.9 to 756 pg/ml (Table 2). Two cytokines (IFN-γ and IL-13) were excluded from further analysis because more than a third of the concentrations were below the lower limit of quantification (43.4% and 34.1%, respectively). A quantifiable concentration was detected in more than 90% of the samples for the remaining cytokines. In total, 7 out of 10 cytokines, including IL-1α, IL-1β, IL-4, IL-5, IP-10, TNF-α, and VEGF-A, showed higher median concentrations in adolescents than in children. IL-5 was the only cytokine that was significantly higher in adolescents. The median concentrations of IL-6 and IL-8 were higher in children, whereas that of IL-10 was similar in both groups.

### Dental examinations

Dental examinations revealed the presence of washable plaque in 56.2%, caries in 38.3%, and gingivitis in 27.3% of the participants (Table 1). Overall, plaque, caries, or gingivitis was not detected in 30.5% of the participants. Gingivitis was more frequent in adolescents (41.2%) than in children (18.2%). By contrast, caries and washable plaque were observed more frequently in children than adolescents (49.4% vs. 21.6% and 66.2% vs. 41.2%, respectively). Supplementary Table 1 presents the characteristics and findings of the oral examinations in participants with and without gingivitis. Participants with gingivitis were significantly older than those without gingivitis (12.8 vs. 9.9 years; p < 0.001); moreover, they presented with increased saliva quantity and flow rate.

Supplementary Table 2 presents the characteristics and clinical profiles of participants with and without caries. The group with caries had a mean age of 8.8 ± 3.9 years; gingivitis was observed in 24.5% and washable plaque in 77.6% of the participants. Both saliva quantity and flow rate were decreased in those with caries.

### Influence of age, sex, dental health, and salivary flow rate on cytokine concentration

#### Age

**Figure 1:**
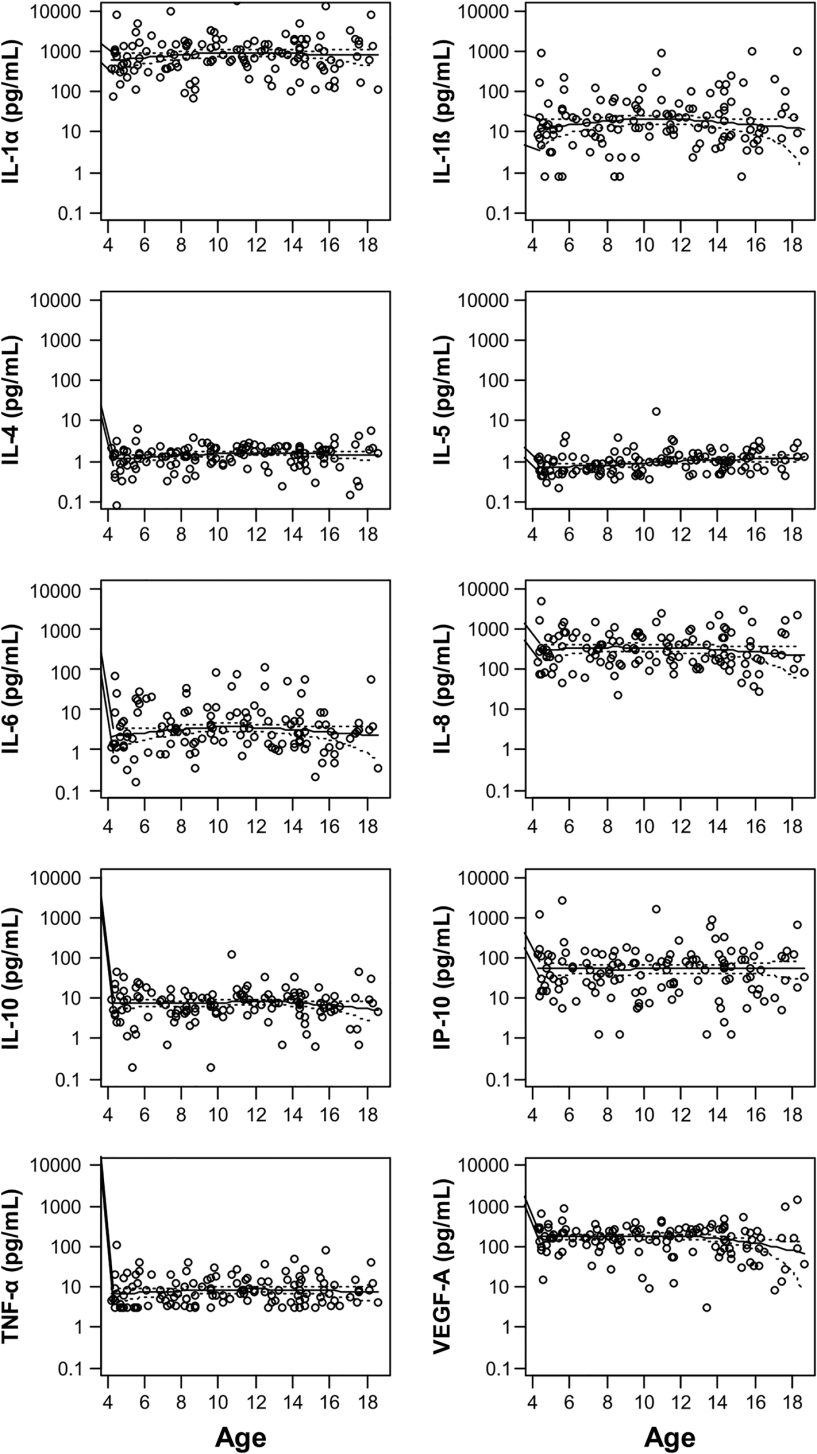
Graphs showing the concentrations of the cytokines across the years (age). A fixed log scale ranging from 0.1 pg/ml to 10 μg/ml was used. The local polynomial regression lines (loess function, degree of smoothing, 2/3) with 95% confidence limits are shown.

The concentrations of all the cytokines were stable over the years, as indicated by the local polynomial regression lines (Fig. 1). Linear regression models with a nested design were calculated to quantify the changes by age in children and adolescents separately (Table 3). The regression models were adjusted for sex, gingivitis, and salivary flow rate. In children, significant annual increases of 11.6%, 15.4%, 8.7%, and 9.3% were observed in IL-1α, IL-1β, IL-4, and IL-5, respectively. Although IL-1β was also substantially increased in adolescents by 22.7% per year, this was only a trend. Similarly, IL-5, and IL-6 demonstrated increasing trends in adolescents and children, respectively, per year.

**Table 3:** Relative annual changes in the cytokine concentration (ratio per year of age).

Cytokine	Ratios (95% CI) per year of age in children; p-value	Ratios (95% CI) per year of age in adolescents; p-value
IL-1α	1.116 (1.006, 1.238); p = 0.039	1.104 (0.933, 1.305); p = 0.25
IL-1β	1.154 (1.002, 1.329); p = 0.048	1.227 (0.972, 1.549); p = 0.085
IL-4	1.087 (1.012, 1.169); p = 0.023	1.025 (0.910, 1.155); p = 0.68
IL-5	1.093 (1.028, 1.162); p = 0.005	1.092 (0.987, 1.208); p = 0.086
IL-6	1.112 (0.982, 1.260); p = 0.093	0.980 (0.798, 1.203); p = 0.84
IL-8	1.023 (0.922, 1.134); p = 0.67	1.036 (0.873, 1.228); p = 0.69
IL-10	1.057 (0.958, 1.167); p = 0.27	1.002 (0.851, 1.179); p = 0.99
IP-10	0.962 (0.833, 1.111); p = 0.59	0.944 (0.748, 1.191); p = 0.62
TNF-α	1.039 (0.961, 1.123); p = 0.33	1.059 (0.932, 1.204); p = 0.38
VEGF-A	0.956 (0.867, 1.055); p = 0.37	0.929 (0.790, 1.093); p = 0.37

A separate linear regression model was performed for each cytokine. The models had a nested design to receive separate estimates for children and adolescents and were further adjusted for sex, gingivitis, caries, and salivary flow rate.

#### Sex

Generally, no significant differences in cytokine concentrations between males and females were observed by linear regression analysis (see Supplementary Table 3). Considering the effect sizes, the concentrations of 8 out of 10 cytokines (VEGF-A, IL-1β, IL-4, IL-5, IL-6, IL-10, IP-10, and TNF-α) were slightly higher in males, with the highest difference seen in IL-5 (39%); alternatively, the concentrations of IL-1α and IL-8 were marginally higher in females.

#### Gingivitis

The concentrations of IL-1β, IL-6, IL-8 and IL-10 were elevated in the presence of gingivitis (Supplementary Table 1). These findings were confirmed by the linear regression models (Supplementary Table 3), where a 2.0, 2.5, 1.6, and 1.6-fold higher concentration was observed for IL-1β, IL-6, IL-8, and IL-10, respectively.

#### Salivary flow rate

The analysis of the cofactors in the linear regression models revealed that salivary flow rate had the most significant effect on the cytokine concentrations (see Supplementary Table 3). Except for IL-4, IL-5, and IP-10, the concentrations of all other measured cytokines were significantly reduced with the increase in the salivary flow rate. The greatest reduction in cytokine concentration per flow rate increase of 1 ml/min was seen for IL-1β (− 59%), IL-6 (− 50%), and IL-1α (− 47%).

#### Caries

Caries appeared to have a minor impact on cytokine concentration, as indicated in Supplementary Tables 2 and 3.

## Discussion

This is the first study to present a comprehensive cytokine profile of children and adolescents, particularly in relation to age, sex, and oral health status. In comparison, former studies investigating cytokines in healthy patients included a smaller number of patients^22–26^, measured fewer cytokines^1,27–29^ or examined patients belonging to a lower age range^1,22,25,28,30–32^.

As quantified using linear regression models, the concentration of the salivary cytokines demonstrated a slight increase with the increase in age in both children and adolescents. This finding is in line with the increase in cytokine concentrations in blood, as reported by Decker et al.^1^. In their study comprising 271 healthy children aged 0–12 years, age-dependent increases in the concentrations of IL-10, IL-6, IL-4, and IL-2 (in unstimulated samples); IL-4, IFN-γ, and TNF-α (in stimulated samples); and IL-2, IL-6, and IP-10 (in C. albicans-stimulated samples) were observed. Similarly, age-dependent increases in the concentrations of TNF-α, IFN-γ, IP-10, and IL-12p70 were reported in stimulated blood samples from children (0–12 months; n = 30) and healthy adults (n = 30)^33^. Stowe et al.^34^, who measured the plasma cytokine profiles in 1411 people aged 25–91 years, observed an increase in the level of IL-6 with aging. However, the findings of the present study were not in accordance with those reported by Riis et al.^35^, who measured the concentrations of cytokines in the saliva and serum of 114 healthy adolescent girls; the concentrations of IFN-γ, IL-1β, IL-2, IL-6, IL-8, IL-10, IL-12p70, and TNF-α were found to be lower in older girls. This discrepancy in results may be attributed to the differences in the study samples and demographics and the potential influence of a smoking habit in the study by Riis et al.

Gingivitis is a common oral finding associated with local inflammation^36^. In the current study, gingivitis was associated with an increase in the concentration of all the cytokines. Santos et al. reported similar findings in unstimulated saliva from 71 children with cerebral palsy, wherein gingival inflammation was related to the increased concentrations of the cytokines (IL-1β, IL-6, IL-8, and TNF-α)^19^. By contrast, Belstrøm et al. reported negative correlations with the salivary cytokine concentrations of IL-1β, VEGF, IL-8, IL-1ra, and MCP-1 in patients with early gingivitis^18^. There is a dearth of studies on the association between cytokine concentration and gingivitis; however, studies on oral inflammatory conditions, such as mucositis, periodontitis, and peri-implantitis, have been published. For instance, different proinflammatory cytokines are thought to play essential roles in the pathogenesis of oral mucositis^10^, and the severity of the disease has been shown to correlate with the intensity of the production of these cytokines^37^. Furthermore, the concentrations of IL-6, TNF-α^10^, and buccal TNF-α (oncological patients)^11^ are reported to be increased in mucositis. Ertugrul et al. demonstrated that the severity of periodontitis correlated with the expression level of IL-8^38^. Liskmann et al. reported elevated IL-6 and IL-10 levels in patients with peri-implantitis^39^. The elevated concentration of IL-6 in children with gingivitis in the current study likely mirrors the underlying pathophysiology of local inflammation. The more frequently observed gingivitis in adolescents could be explained by the fact that there is an age-related tendency for the development of gingivitis, with children showing a lower severity of gingivitis than adults. Certain oral findings are rare or even absent in our study population. This was to be expected, as these findings are not likely to be seen in healthy young individuals.

To the best of our knowledge, the correlation between oral inflammation and salivary flow rate has not been described thus far. Saliva is important for the maintenance of oral health^40^. The concentrations of all cytokines examined in this study decreased with the increase in the salivary flow rate. This observation could be explained by the dilution of the cytokines due to the high salivary flow rate; alternatively, the antiinflammatory and antibiotic components in the saliva might exert a protective effect against oral inflammation, which has been reported recently^41,42^. Furthermore, the salivary flow rate is reported to be reduced in chronic periodontitis, a subcategory of oral inflammation^43^.

Because of the limited number of participants in this study, it was not possible to perform a complete logistic regression analysis with all the cytokines integrated as cofactors without the problem of overfitting. Therefore, separate linear regression models were used to predict each cytokine. Longitudinal study designs using larger samples and more diverse populations are needed to further characterize the cytokine expression and its association with the altered conditions in the oral cavity.

## Conclusion

In summary, the concentrations of the cytokines were correlated with age, sex, and gingivitis in this study. The proinflammatory cytokines IL-6 and IL-1β were prominently associated with gingivitis, suggesting that the levels of these cytokines could predict or confirm oral inflammation. Furthermore, the salivary flow rate influenced the cytokine concentrations; however, the interpretation of this finding remains unclear. The findings of this study confirm the feasibility of measuring salivary cytokines. Nonetheless, additional studies are required to ascertain the prognostic significance of cytokines in oral health in the pediatric population.

## Supplementary Information

Supplementary Tables.

## Data Availability

The data set from the study are held securely in coded form at the Ambulantes Studienzentrum (ASZ) of the University Children’s hospital of Basel. The data underlying this article will be shared on reasonable request to the corresponding authors after granting prespecified criteria for confidential access.
