# Supplementary material for: Associations between salivary cytokines and oral health, age, and sex in healthy children
Source: Sci Rep. 2022 Sep 26;12:15991. doi: 10.1038/s41598-022-20475-2 (PMC9512800; doi:10.1038/s41598-022-20475-2)
Supplement: Supplementary file 1 — Supplementary Tables. [file 41598_2022_20475_MOESM1_ESM.docx]

**Supplementary Table 1. Characteristics, clinical profiles, and cytokine concentrations in the presence and absence of gingivitis.**

|  | **Gingivitis**  **n = 35 (out of 128)** | **No gingivitis**  **n = 93 (out of 128)** | **p-value** |
| --- | --- | --- | --- |
| Males/Females^a^ | 18/17 (51.4/48.6) | 47/46 (50.5/49.5) | 1 |
| Age^b^ | 12.8 (3.4) | 9.9 (4.2) | <0.001 |
| Children/Adolescents^a^ | 14/21 (40/60) | 63/30 (67.7/32.3) | 0.008 |
| **Stomatological and dental findings** |  |  |  |
| Caries^a^ | 12 (34.3) | 37 (39.8) | 0.71 |
| Washable plaque^a^ | 29 (82.9) | 43 (46.2) | <0.001 |
| Xerostomia^a^ | 0 (0) | 0 (0) | n.e. |
| Candidiasis^a^ | 1 (2.9) | 0 (0) | 0.27 |
| Decayed teeth^c^ | 0 (0, 1) | 0 (0, 2) | 0.96 |
| Missing teeth^c^ | 0 (0, 0) | 0 (0, 1) | 0.092 |
| Filled teeth^c^ | 1 (0, 2.8) | 2 (0, 5) | 0.10 |
| Any mucosal changes^a^ | 1 (2.9) | 5 (5.4) | 1 |
| Aphthae^a^ | 1 (2.9) | 1 (1.1) | 0.47 |
| Saliva total quantity (ml) ^c^ | 5.5 (3.6, 10.2) | 3.8 (2.3, 5.7) | 0.007 |
| Saliva flow rate (ml/min) ^b^ | 1.3 (0.8) | 0.9 (0.7) | 0.007 |
| **Cytokines, pg/ml** |  |  |  |
| IL-1α^c^ | 989 (576, 1407) | 734 (381, 1477) | 0.39 |
| IL-1β^c^ | 23.4 (11.4, 61.1) | 13.2 (7.3, 32.2) | 0.040 |
| IL-4^c^ | 1.4 (1, 2.2) | 1.5 (0.9, 2) | 0.65 |
| IL-5^c^ | 1 (0.6, 1.4) | 0.9 (0.6, 1.3) | 0.64 |
| IL-6^c^ | 4.4 (2.3, 23.2) | 2.6 (1.2, 5.5) | 0.008 |
| IL-8^c^ | 360 (200, 767) | 257 (122, 590) | 0.080 |
| IL-10^c^ | 10.4 (5.9, 12.2) | 7 (4.1, 10.6) | 0.016 |
| IP-10^c^ | 77.4 (18.2, 127) | 49.9 (16.8, 92.2) | 0.22 |
| TNF-α^c^ | 9 (6.3, 16.1) | 6.4 (4.2, 12.1) | 0.084 |
| VEGF-A^c^ | 176 (87, 262) | 163 (91.4, 249) | 0.52 |

n.e. not estimatable

^a^ The values are given as the frequency (and proportion)

^b^ The values are given as the mean (and the standard deviation)

^c^ The values are given as the median (and the interquartile range)

**Supplementary Table 2.** **Characteristics, clinical profiles, and cytokine concentrations in the presence and absence of caries.**

|  | **Caries**  **n = 49 (out of 128)** | **No caries**  **n = 79 (out of 128)** | **p-value** |
| --- | --- | --- | --- |
| Males/Females^a^ | 28/21 (57.1/42.9) | 37/42 (46.8/53.2) | 0.34 |
| Age^b^ | 8.8 (3.9) | 11.9 (4.0) | <0.001 |
| Children/Adolescents^a^ | 38/11 (77.6/22.4) | 39/40 (49.4/50.6) | 0.003 |
| **Stomatological and dental findings** |  |  |  |
| Gingivitis^a^ | 12 (24.5) | 23 (29.1) | 0.71 |
| Washable Plaque^a^ | 38 (77.6) | 34 (43) | <0.001 |
| Xerostomia^a^ | 0 (0) | 0 (0) | n.e. |
| Candidiasis^a^ | 1 (2) | 0 (0) | 0.38 |
| Decayed teeth^c^ | 2 (1, 6) | 0 (0, 0) | <0.001 |
| Missing teeth^c^ | 0 (0, 1) | 0 (0, 0) | 0.053 |
| Filled teeth^c^ | 2 (0, 4) | 1 (0, 4) | 0.36 |
| Any mucosal changes^a^ | 3 (6.1) | 3 (3.8) | 0.67 |
| Aphthae^a^ | 1 (2.0) | 1 (1.3) | 1 |
| Saliva total quantity (ml) ^c^ | 3.3 (2.4, 6.1) | 4.5 (2.8, 7.0) | 0.054 |
| Saliva flow rate (ml/min) ^b^ | 0.9 (0.7) | 1.1 (0.7) | 0.057 |
| **Cytokines, pg/ml** |  |  |  |
| IL-1α^c^ | 663 (402, 1445) | 803 (404, 1473) | 0.59 |
| IL-1β^c^ | 14.2 (7.4, 36.6) | 18.2 (8.1, 39.7) | 0.72 |
| IL-4^c^ | 1.3 (0.8, 1.8) | 1.5 (0.9, 2.1) | 0.30 |
| IL-5^c^ | 0.8 (0.6, 1.1) | 1.1 (0.6, 1.4) | 0.020 |
| IL-6^c^ | 3.1 (1.4, 8.6) | 2.9 (1.4, 7.1) | 0.61 |
| IL-8^c^ | 297 (187, 615) | 265 (132, 585) | 0.30 |
| IL-10^c^ | 8.1 (4.6, 14.2) | 7.3 (3.8, 11.5) | 0.23 |
| IP-10^c^ | 51.2 (14.9, 103) | 53.5 (19, 106) | 0.90 |
| TNF-α^c^ | 6.7 (3.9, 15.1) | 8.1 (4.5, 12.9) | 0.40 |
| VEGF-A^c^ | 164 (101, 249) | 162 (86.1, 257) | 0.74 |

n.e. not estimatable

^a^ The values are given as the frequency (and proportion)

^b^ The values are given as the mean (and the standard deviation)

^c^ The values are given as the median (and the interquartile range)

**Supplementary Table 3.** **Effect sizes of the cytokine concentrations due to the cofactors included in the regression models.**

| **Cytokine** | **Cofactors** | **Ratios (95% CI); p-value** |
| --- | --- | --- |
| IL-1α | Sex: male vs female  Gingivitis: yes vs no  Caries: yes vs no  Flow rate | 0.781 (0.546, 1.116); p = 0.17  1.259 (0.823, 1.925); p = 0.25  1.045 (0.704, 1.551); p = 0.83  0.526 (0.391, 0.707); p <0.001 |
| IL-1β | Sex: male vs female  Gingivitis: yes vs no  Caries: yes vs no  Flow rate | 1.066 (0.654, 1.737); p = 0.80  1.980 (1.106, 3.545); p = 0.022  1.099 (0.641, 1.882); p = 0.73  0.414 (0.277, 0.619); p <0.001 |
| IL-4 | Sex: male vs female  Gingivitis: yes vs no  Caries: yes vs no  Flow rate | 1.235 (0.963, 1.584); p = 0.096  1.021 (0.758, 1.373); p = 0.89  1.008 (0.766, 1.327); p = 0.95  0.928 (0.756, 1.140); p = 0.47 |
| IL-5 | Sex: male vs female  Gingivitis: yes vs no  Caries: yes vs no  Flow rate | 1.391 (1.126, 1.718); p = 0.002  1.025 (0.797, 1.319); p = 0.85  0.901 (0.714, 1.138); p = 0.38  0.954 (0.802, 1.136); p = 0.60 |
| IL-6 | Sex: male vs female  Gingivitis: yes vs no  Caries: yes vs no  Flow rate | 1.014 (0.660, 1.557); p = 0.95  2.451 (1.469, 4.090); p = 0.001  1.289 (0.803, 2.070); p = 0.29  0.497 (0.349, 0.708); p <0.001 |
| IL-8 | Sex: male vs female  Gingivitis: yes vs no  Caries: yes vs no  Flow rate | 0.836 (0.585, 1.194); p = 0.32  1.630 (1.065, 2.494); p = 0.025  1.212 (0.818, 1.796); p = 0.33  0.652 (0.486, 0.875); p = 0.005 |
| IL-10 | Sex: male vs female  Gingivitis: yes vs no  Caries: yes vs no  Flow rate | 1.042 (0.741, 1.466); p = 0.81  1.646 (1.096, 2.472); p = 0.017  1.533 (1.053, 2.233); p = 0.026  0.749 (0.566, 0.992); p = 0.044 |
| IP-10 | Sex: male vs female  Gingivitis: yes vs no  Caries: yes vs no  Flow rate | 1.534 (0.936, 2.514); p = 0.089  1.242 (0.690, 2.236); p = 0.47  1.229 (0.712, 2.124); p = 0.46  1.041 (0.692, 1.568); p = 0.84 |
| TNF-α | Sex: male vs female  Gingivitis: yes vs no  Caries: yes vs no  Flow rate | 1.175 (0.899, 1.537); p = 0.24  1.367 (0.992, 1.882); p = 0.056  0.892 (0.664, 1.200); p = 0.45  0.723 (0.580, 0.902); p = 0.004 |
| VEGF-A | Sex: male vs female  Gingivitis: yes vs no  Caries: yes vs no  Flow rate | 1.071 (0.762, 1.504); p = 0.69  1.446 (0.964, 2.170); p = 0.075  0.967 (0.665, 1.408); p = 0.86  0.759 (0.573, 1.005); p = 0.054 |

Additional effect sizes of the cofactors included in the regression analysis summarised in Table 3. The effect size of the cytokines for flow rate is ratio per one unit increase (1 ml/min) of flow rate.
